# Foreign body in the esophagus: a review

**DOI:** 10.1590/S1516-31802006000600010

**Published:** 2006-11-01

**Authors:** Omer Ashraf

**Keywords:** Foreign bodies, Esophagus, Review literature, Endoscopy, Observation, Cuerposextraños, Esófago, Literatura de revisión, Endoscopía, Observación

## Abstract

Foreign body in the esophagus is a common emergency presentation. The approach towards a patient with a foreign body in the esophagus comprises a thorough history and systematic examination followed by relevant investigations. However, there is considerable debate over the most appropriate treatment option for such patients. This review aims to develop a comprehensive approach towards patients presenting with foreign body ingestion by developing clinical practice guidelines. These guidelines address not only the initial evaluation of the patient but also the various management alternatives and their advantages, limitations and applicability in various scenarios, based upon a review of the literature.

## RESUMEN

El cuerpo extraño en el esófago es una presentación de la emergencia. Acñrquese hacia un paciente con un cuerpo extraño esofágico comprende una historia completa y examen sistemático seguidos por investigaciones pertinentes. Sin embargo hay el debate considerable sobre la opción del tratamiento más apropiada para tales pacientes. Esta revisión se propone desarrollar un enfoque completo hacia un paciente que presenta con ingestión de cuerpo extraño desarrollando una pauta clínica de la práctica en no apenas la evaluación inicial del paciente pero también dirigiendo las varias alternativas de la administración, sus ventajas y las limitaciones y la aplicabilidad en varios guiones, basado sobre una revisión de la literatura.

## INTRODUCTION

Foreign body (FB) ingestion is an everyday occurrence and a common emergency presentation. Many ingested FBs become impacted, often in the esophagus, and have the potential to cause serious complications, apart from significant distress to the patient and family.

Despite the frequency and seriousness of this issue, there is considerable argument in the literature regarding the best possible approach for dealing with patients with an FB in the esophagus. It is imperative to devise uniform guidelines. This review aims to develop an approach along these lines by taking into account the recent findings in the literature. It begins with an overview of the types of objects usually encountered and their usual impaction sites at the time of presentation, and then formulates an approach towards such patients. Finally, this review covers the various management alternatives and concludes with a standardized overall method for dealing with these patients.

## METHODS

The literature was primarily searched through three databases: PubMed, Literatura Latino-Americana e do Caribe em Ciências da Saúde (Lilacs) and the Cochrane Library of systematic reviews. The terms "esophagus" and "foreign body" were utilized in the search, to obtain the list of relevant articles. Most of the results came from PubMed (58 articles), while Lilacs produced two results and the Cochrane database did not reveal any results of significant relevance. The articles with relevant and significant findings were then adapted and used in writing this review.

### Foreign body ingestion

While some ingested FBs may be aspirated, most are either regurgitated or pass through the gastrointestinal tract without causing any complications. The lodgment site has been found to be influenced by age,^1,2^ FB type^3,4^ and duration of ingestion, as well as certain individual pathological conditions like stricture, stenosis, fistula, etc. Overall, 28-68% of gastrointestinal FBs are found in the esophagus.^5^ The most frequent lodgment site in childreen is at the level of the cricopharyngeus muscle (which is the narrowest part of the esophagus), and in adults it is at the lower esophageal sphincter or at the site of any predisposing lesion.^1,2^ Since most of the presentations are in children^1,2,6-10^ the overall commonest site of FB presentation in the esophagus is in its upper third.^11,12^ The individual characteristics of the ingested body also determine the lodgment site. Large and rigid FBs tend to lodge in the pyriform fossa and esophagus.^13^ While fish bones are usually found in the pharynx,^3^ coins and impacted meat are usually in the proximal and distal parts of the esophagus respectively.^4^ Aspirated objects often consist of nuts or seeds.^3,14,15^

A wide variety of esophageal foreign bodies are seen in clinical practice. Coins are the commonest overall^1,7,16-18^ and the commonest single type in children, while bones comprise the bulk of FBs in adults.^9,11,17,19,20^ Other objects regularly seen include meat, cartilage, dentures, bezoars, fruit stones, toys, batteries and buttons. Among the more dangerous ones are batteries, needles, safety razors, dentures with wires, spring coils and pieces of glass. Factors that predispose towards greater risk of esophageal FB impaction include male gender,^7,21,22^ underlying esophageal stricture,^1,2,5,23,24^ neuromuscular disease (myasthenia *gravis*),^2^ external and mechanical factors, ankylosing spondylitis,^2^ mental retardation, psychiatric illness,5 use of dentures,^15,25^ Chinese methods of cooking and eating,^26-28^ and being a prisoner.^5^ Peptic stenosis is the commonest of these,^29^ but most of these underlying mechanisms are generally only of significant relevance for adults.

### Diagnostic approach towards patients

Patient presentations vary, although dysphagia and odynophagia are the most frequently reported symptoms.^5,9^ Other features that may be present include history of FB ingestion,^9^ presence of persistent FB sensation,^7,20,30^ chest pain,^18^ pooling of saliva,^7,12^ vomiting^1,14^ and regurgitation.^1,5^ There may also be respiratory symptoms of stridor,^31-33^ cough and choking,^14,33^ which are generally found in younger children with chronic FB impaction lasting more than one week.^34^

Despite being less common than nasal and pharyngeal FBs,^3^ esophageal FBs are emergency situations and require timely management because of the potentially life-threatening complications they pose. While the most important step is to establish an airway,^1^ the overall approach towards patients with esophageal FBs comprises a meticulous history, methodical examination and pertinent investigations followed by prompt and appropriate management. The history points towards the diagnosis in most cases and indeed, a clinical history may well be the main indicator for further intervention.^35^ The FB type and the duration and nature of symptoms^7,30^ are all useful indicators regarding the lodgment site and the need for immediate intervention. If the FB is known to be a radiolucent object, this would rule out the use of roentgenographic study as a diagnostic method. Even though esophagoscopy is conducted in most patients with an esophageal FB worldwide, asymptomatic patients with acute ingestion should be followed for spontaneous passage of the FB.^31,36^ However, it should be noted that a negative history does not rule out an FB and a high degree of suspicion should be maintained in children and impaired adults with unexplained compromised respiration.

The next step is a thorough physical examination of the patient. The patient could either be asked to point towards the area of maximum discomfort (or FB sensation) or be asked to swallow to determine the possible site of FB lodgment.^30^ The water-drinking test and positive laryngeal rub both have high sensitivity and specificity for esophageal FBs.^13^ However, adequate visualization of the oral cavity, nasal passages, pharynx and larynx is crucial for ruling out an FB in the upper aerodigestive tract. Tongue depressor, transnasal flexible endoscopy, indirect laryngeal mirror, Mackintosh laryngoscope and flexible pharyngolaryngoscopy may subsequently be utilized. Examination is crucial and indeed is generally reliable for supracricoid FBs. For cricoid and infracricoid bodies, however, further careful monitoring is warranted.^30^

Investigations then follow for further assessment of the patient and are mainly of imaging type. X-ray evaluation is indicated for all patients in whom an esophageal FB is suspected.^5,37^ Lateral and anteroposterior roentgenograms of the neck, along with chest and abdomen x-rays, can be conducted to elicit a radiopaque FB. Barium studies are also useful.^19,35^ In undetected cases^1^ and cases of suspected perforation, computed tomography (CT) scanning should be done.^24^ The presence of even radiolucent objects can be hinted at by air entrapment in the preceding portion of the gut,^7^ although radiological findings are not considered helpful for identification purposes in cases of radiolucent FBs.^38^ Active management is now generally indicated in all cases with symptoms, positive findings at assessment or incomplete examination.^15,39^

### Management

The final and naturally the most critical aspect of dealing with these patients is treatment, and this is the area shrouded in the greatest controversy. Even with the myriad management techniques available today, there still exists significant debate about the appropriate management procedures for patients with esophageal FBs. Various protocols have been advocated around the world with claims of comparable efficacy and safety.

There are a variety of management options available. These include inpatient or outpatient observation, pharmacological therapy, flexible endoscopy, rigid endoscopy, Foley catheter removal, esophageal bougienage, forceps extraction and surgery, apart from a few other innovative practices. While esophagoscopy may be the most popular approach, every technique has its advantages and limitations and the eventual decision is usually a result of personal and local preferences.^40^ It is therefore obligatory to individually appraise each procedure in order to formulate guidelines with some universality.

While some FBs may be aspirated, leading to disastrous complications, particularly in young children,^41,42^ most ingested FBs tend to pass through the gastrointestinal tract spontaneously, and only a fraction require intervention.^43^ The initial approach is therefore, in non-critical cases, to watch and wait for the object to be expelled of its own accord. Observation is generally indicated for asymptomatic patients with a history of nonthreatening FB ingestion over periods of less than 24 hours^31^ and without any respiratory symptoms or history of esophageal disease or surgery.^44^ In scenarios such as these, monitoring can be done on an inpatient or outpatient basis. Outpatient observation has been shown to reduce costs markedly in comparison with the vastly popular endoscopic removal.^45^ Inpatient surveillance, while offering little or no such advantage, has been found to be associated with higher rates of spontaneous coin passage.^45^ Both of these methods have, however, been known to significantly reduce complications in comparison with endoscopy.^45^ While observation is more successful in older children and distal impaction,^31,46^ it should generally be substituted by removal in cases of proximal FB lodgment, inability to breathe or tolerate oral fluids, and positive repeat radiography.^31^ Pharmacological therapy can also be tried, and encouraging results have been obtained from parenteral diazepam and glucagon administration.^4^

The most prevalent therapy for esophageal FBs is endoscopy. This is both a diagnostic and a management method and is generally recommended for most patients with history of FB ingestion.^1,8,29,37^ The two common variants, flexible and rigid endoscopy, are complementary and available in most tertiary care units today. Rigid endoscopy may be less expensive,^47^ better suited for proximal and sharp objects,^30^ and predominant in many regions of the world. On the other hand, forward-viewing flexible panendoscopy can be performed under local anesthesia, is more suited for intrathoracic objects,^30^ with equal efficacy^38^ and lower complication rates,^48^ and is now the instrument of choice for managing FBs in most tertiary medical centers as well as in community hospitals.^38,47^ The complications typically encountered include perforation, laceration, abscess formation and mediastinitis.^12,30,49^ The limitations on endoscopic coin removal include greater expense and time consumption, the need for endotracheal intubation, anesthesia and an operating suite, postprocedural hospitalization and greater complication rates than those experienced with other contemporary techniques. In spite of all this, endoscopy is still widely regarded as the most successful and reliable technique for FB removal.

Foley catheter extraction involves passing a balloon catheter distally to the ingested object, inflating the balloon, and withdrawing the catheter and the ingested object proximally. This is usually conducted under fluoroscopic guidance, and has been established as a relatively safe^50,51^ and cost-effective procedure.^40,51^ It is generally known for its usefulness in removing recently ingested and proximally located blunt objects.^48,52^ It has been shown to be effective even without the usual sedation^52^ and fluoroscopic guidance^53^ and is recommended as the treatment of choice for retained coins in children who do not show signs of significant esophageal edema that would causing tracheal impairment.^50^ However there are certain contraindications for the use of the Foley catheter technique, and these include FB ingestion more than 24 hours before intervention or at an unknown earlier time, prior esophageal stricture or surgery, signs and symptoms of marked esophageal obstruction, stridor or compromised respiration.^54-57^ Its limitations include anesthesia, intravenous access and fluoroscopic guidance. Taking more than five minutes to perform fluoroscopy is associated with a low probability of success^21^ and, under such circumstances, fluoroscopy can be followed by endoscopic removal.

Another method similar in its attributes to the Foley procedure is the technique of esophageal bougienage. This is performed on an unsedated patient sitting upright, and involves the passage of a single bougie dilator from mouth to stomach. This leads to advancement of the FB into the stomach, from where it is most likely to pass spontaneously onwards. Because this technique does not require anesthesia or sedation, it is best indicated in situations where a smooth, round object could readily be mechanically advanced distally to the stomach with little risk of complications.^58^ Bougienage is extremely cost effective^40,51^ and almost free of complications,^45^ but its widespread has been restricted by limited publicity and prerequisites in patient selection akin to those for the balloon catheter method. Such requirements include recent ingestion with no respiratory involvement, no history of esophageal disease or surgery,^51^ and normal esophageal wall strength, distensibility and lumenal diameter.^59^ Further research is necessary in this direction for esophageal bougienage approach to become firmly established on an international scale.

Magill forceps have also been found to be a possible method for removing coins from the upper esophagus or just below the cricopharyngeus.^32^ This method is minimally invasive and quick, and can be used in children with respiratory distress (because the airway is secure), or when the duration of coin impaction is indeterminate, or there has been previous esophageal surgery.^32^ Before going ahead with this technique, the lodgment should be radiographically confirmed and it should be confirmed that there is no clinical evidence of perforation.^32^

Surgery is rarely performed,^5,18,24,47^ but is relatively successful. It is indicated in cases of perforation, other complications and failure to remove the coin by other, preceding techniques.^60^

## CONCLUSION

The clinical practice guidelines that incorporate most of these principles, as illustrated by this literature review, would therefore be as follows.

The initial approach towards a patient with an esophageal FB demands urgent assessment of the respiratory status and establishment of an airway. Apart from this, the history is the most important part of the early evaluation. Examination should be followed by roentgenographic study. Asymptomatic patients with an acute presentation and lack of respiratory impairment can be followed on an outpatient or inpatient basis, depending on the clinical picture. In the event of non-resolution of symptoms, positive findings from repeat radiography and chronic impaction, intervention should be sought without delay. In selected cases, balloon extraction or bougienage may be attempted, considering their low cost and lack of complications and the fact that esophagoscopy can, in any event, be performed subsequently. Rigid pharyngoesophagoscopy may be used for removing sharp objects above the thoracic inlet, while flexible endoscopy should be preferred for intrathoracic FBs. Surgery remains the last alternative.

FBs in the esophagus will continue to be a common emergency. Even though better community education and parent teaching programs might be of some value, it is unlikely that there will be a significant reduction in the incidence of the most common reason for emergency endoscopy: esophageal FBs. The best approach towards this problem lies in developing standard guidelines for dealing with this situation, a hope that can only turn into reality through further evidence-based medicine along these lines and general publicity.
